# Cardiovascular risk management in patients with severe mental illness or taking antipsychotics: A qualitative study on barriers and facilitators among dutch general practitioners

**DOI:** 10.1080/13814788.2022.2092093

**Published:** 2022-07-07

**Authors:** Kirsti Jakobs, Latoya Lautan, Peter Lucassen, Joost Janzing, Jan van Lieshout, Marion C. J. Biermans, Erik W. M. A. Bischoff

**Affiliations:** a Department of Primary and Community Care, Radboud Institute for Health Sciences, Radboud University Medical Centre, Nijmegen, The Netherlands; b Department of Psychiatry, Radboud Institute for Health Sciences, Radboud University Medical Centre, Nijmegen, The Netherlands; c Department IQ Healthcare, Radboud Institute for Health Sciences, Radboud University Medical Centre, Nijmegen, The Netherlands

**Keywords:** Primary care, qualitative research, severe mental illness, antipsychotics, cardiovascular risk

## Abstract

**Background:**

Patients with severe mental illness (SMI) or receiving treatment with antipsychotics (APs) have an increased risk of cardiovascular disease. Cardiovascular risk management (CVRM) increasingly depends on general practitioners (GPs) because of the shift of mental healthcare from secondary to primary care and the surge of off-label AP prescriptions. Nevertheless, the uptake of patients with SMI/APs in CVRM programmes in Dutch primary care is low.

**Objectives:**

To explore which barriers and facilitators GPs foresee when including and treating patients with SMI or using APs in an existing CVRM programme.

**Methods:**

In 2019, we conducted a qualitative study among 13 Dutch GPs. During individual in-depth, semi-structured interviews a computer-generated list of eligible patients who lacked annual cardiovascular risk (CVR) screening guided the interview. Data was analysed thematically.

**Results:**

The main barriers identified were: (i) underestimation of patient CVR and ambivalence to apply risk-lowering strategies such as smoking cessation, (ii) disproportionate burden on GPs in deprived areas, (iii) poor information exchange between GPs and psychiatrists, and (iv) scepticism about patient compliance, especially those with more complex conditions. The main facilitators included: (i) support of GPs through a computer-generated list of eligible patients and (ii) involvement of family or carers.

**Conclusion:**

This study displays a range of barriers and facilitators anticipated by GPs. These indicate the preconditions required to remove barriers and facilitate GPs, namely adequate recommendations in practice guidelines, improved consultation opportunities with psychiatrists, practical advice to support patient adherence and incentives for practices in deprived areas.

KEY MESSAGES

- Implementation of CVRM in patients with SMI by GPs may be hindered by a lack of knowledge about the additional cardiovascular risk, stigma towards this patient group, and a high workload.
- A supportive list with eligible patients and support by psychiatrists and caregivers may facilitate GPs with the implementation.

## Introduction

A meta-analysis of 92 studies revealed that people with mental disorders have an elevated cardiovascular risk (CVR) [1]. Patients with a severe mental illness (SMI), including schizophrenia, bipolar disorder, and non-organic psychosis, had the highest risk [1]. A Dutch study showed the all-cause mortality rate was four to five times higher in people with SMI than in the general population, mainly due to cardiovascular mortality [2]. The high CVR in patients with SMI may be related to stress, an unhealthy lifestyle, addictions and distance to healthcare [3,4]. Another essential factor in this elevated risk is the adverse metabolic effects of taking atypical antipsychotic (AP) medications [5]. The European guideline on cardiovascular disease prevention updated after our study was completed, recommends intensified attention and support to improve adherence to lifestyle changes and drug treatment for these patients [6].

The global rates of CVR management (CVRM) remain low in patients with SMI or those using Aps, suggesting an undertreatment of CVR in these patients [7,8]. In the Netherlands, the prevalence of patients with SMI and those who use APs in the Netherlands is estimated at 1.5% [8]. GPs are increasingly responsible for CVRM in patients with SMI or who use APs as a result of the governmentally generated shift of mental healthcare from secondary to primary care [9,10], as well as the growing number of off-label prescriptions of APs either initiated or continued by the GP [11]. Approximately 40–75% of current AP prescriptions are for off-label uses, particularly for anxiety and insomnia [11]. Both patients and GPs with expertise in mental health issues believe that CVRM should be delivered by the GP rather than psychiatrists [12,13].

Dutch GPs are well organised in so-called ‘primary care co-operatives’, which aim to improve chronic disease management for patients with diabetes mellitus, cardiovascular diseases, or high CVR [14]. GPs delegate chronic disease management to specialised nurses who help patients improve their lifestyles and provide CVR-lowering medication. In this system, patients are proactively invited for a check-up at least yearly. Including patients is based on risk categories in the multidisciplinary CVRM guideline, which does not explicitly mention patients with SMI or those taking APs [15]. Due to a regional agreement with the health insurance companies, GPs connected to a co-operative in the eastern part of the Netherlands can include all patients with SMI or using APs in the chronic disease management programme. However, 4 years after the initiation of this agreement, the attendance of this patient group remains low.

Previous studies explored the low rates of CVR screening for patients with SMI or using APs. First, guidelines are ambivalent about whose role it is to screen and optimise CVR [12]. Secondly, healthcare professionals are inconsistent in their approach and sometimes negatively perceive psychiatric patients, particularly regarding smoking cessation [16,17]. Thirdly, patient access to primary care is hindered by limited help-seeking behaviour, psychological barriers, and poor understanding of preventing physical illness [17]. These studies were chiefly conducted through questionnaires or focus groups among healthcare professionals, family members, or patients. They were performed in countries with different healthcare systems, often before the implementation of chronic disease management programmes in primary care. The process of proactively inviting patients for CVRM in primary care starts with the GPs’ willingness to do so. It is therefore vital to gain insight into the views of GPs. We aimed to explore which barriers and facilitators GPs perceive when including and treating patients with SMI or using APs in an existing CVRM programme.

## Methods

### Study design

We performed a qualitative study based on interviews with GPs about their views on and experiences with CVRM in patients with SMI or AP use. We used in-depth, face-to-face, semi-structured interviews to examine the scope of the factors involved. To identify these factors, we started every interview with an open approach followed by a phase where we used an interview guide (see Supplementary Material) based on the Consolidated Framework Implementation Research (CFIR) model [18]. This framework includes the following domains: intervention characteristics, characteristics of individuals, inner and outer setting of the general practice, and implementation process. We reported this study according to the COREQ guidelines [19].

### Selection of GPs

Fourteen GPs were approached from ‘Onze Huisartsen’, a regional primary care co-operative for chronic disease management in the eastern part of the Netherlands. In this region, local financial agreements make the CVRM programme accessible for patients with SMI or AP use. We based the selection of GPs for the interviews on purposive sampling to obtain as much variation in GP experiences as possible. The research group (all authors) discussed and agreed on the rationale for ten relevant characteristics of GPs and their practices as shown in Table 1. Considering various characteristics that might influence the opinion of the GP, such as ‘size of the organisation’, ‘socio economic status of patient population’, and ‘collaboration with different (regional) mental healthcare providers’.

**Table 1.** Characteristics of participating GPs (*n* = 13).

Axis of diversity	Participants	*N*
Gender	Male	6
	Female	7
Age	≤45	5
	>45	8
Association with university/GP training	Academic practice	7
	Non-academic practice	6
Size of organisation	≤10 employees	8
	>10 employees	5
Location of organisation	Rural	4
	Urban	6
	Combined	3
Socio economic status (SES) of patient population	SES lower than average	6
	SES average or higher	7
Proportion of elderly in patient population	Elderly population higher than average	5
	Elderly population average or lower	8
Collaboration with different mental healthcare providers	Main collaboration with provider X	8
	Main collaboration with provider Y	3
	Main collaboration with provider Z	2
Semi-institutionalised patients registered in the practice	Yes	6
	No	7
Proportion of new migrants (raised abroad) in patient population	New migrant population higher than average	6
	New migrant population average or lower	7

### Procedure

GPs were invited by telephone to join the study. All but one, who was too busy, agreed to an interview. We conducted all interviews with guidance from a list of patients registered in the GP’s practice. This list included all patients with SMI and/or those using APs who were not participating in the chronic disease management programme. The list was generated from electronic medical files, including patients with the following ICPC codes [20]: P72 (schizophrenia), P73 (affective psychoses), P73.02 (bipolar disorder), and P98 (non-specific psychoses), and the ATC code [21] N05A (unless prescribed for dementia or delirium).

GPs used the list during the interview as a tool to conceptualise what might be facilitators and barriers, using their professional knowledge and broad experiences, without revealing privacy-sensitive data. We started the interviews with an open, inductive approach to give GPs ample opportunity to bring up factors they considered necessary. The list of patients supported this phase in the interview. Our interview guide was deductively composed of CFIR framework elements and was used in the last phase of the interview. GPs would often go back to the list in this phase to illustrate their answers. We estimated 10–11 interviews would be needed to identify the scope of relevant factors. We planned two additional interviews until no new factors were found and saturation was achieved.

### Interviews

Either one researcher (KJ or LL) or both conducted the interviews between April 2019 and October 2019 in general practice setting. The interviews lasted between 30 and 60 min. The researchers are female, had prior training in qualitative methodology, and tended to approach the participants with an open unjudgmental attitude. Researcher KJ is a GP and works for the participating primary care co-operative as a medical advisor on cardiovascular diseases. Researcher LL is a master student. The data analysis started after the first five interviews and data collection and analysis were alternated. Thus, the following interviews could address questions that emerged during the analysis.

### Data analysis

We digitally recorded all interviews, transcribed and imported them into Atlas.ti8 for further analysis. Thematic analysis, as described by Braun and Clarke [22], was chosen. We aimed to display the whole range of motivations, experiences, and meaning of different GPs and argument what should be considered if GPs are asked to include and treat patients with SMI or APs in the CVRM programme. The analysis was performed as follows: (1) repeated review of the transcripts to gain insights into the contents(KJ and LL), (2) independent open coding of the transcripts (KJ and LL), (3) discussion of codes to identify the underlying ideas or assumptions(all), and (4) merging codes from categories into themes reflecting the barriers and facilitators. Concepts of themes were refined by going back and forth through the data. This refinement process was used to provide a clear sense of the scope and diversity of each theme. The research group had extensive discussions about the content of the themes and categories. The research group discussed the data in fortnightly meetings to improve the validity, and all agreed on the final categories and themes.

### Ethics

This study was conducted according to Dutch legislation on privacy and the declaration of Helsinki. There were no conflicts of interest. Ethical approval for this study was asked for but not considered necessary by the local Medical Research Ethics Committee Arnhem/Nijmegen (file 2019-5186). We audio-recorded the verbally obtained informed consent from all GPs and their interviews were pseudonymised.

## Results

The characteristics of the thirteen GPs who participated in the interviews are shown in Table 1.

Our analysis yielded four themes and 12 categories (see Supplementary Figure S1). The findings are presented in relation to the four themes and are illustrated by quotes. An overview of the main barriers and facilitators for each theme are shown in Table 2.

**Table 2.** Barriers and facilitators for each theme.

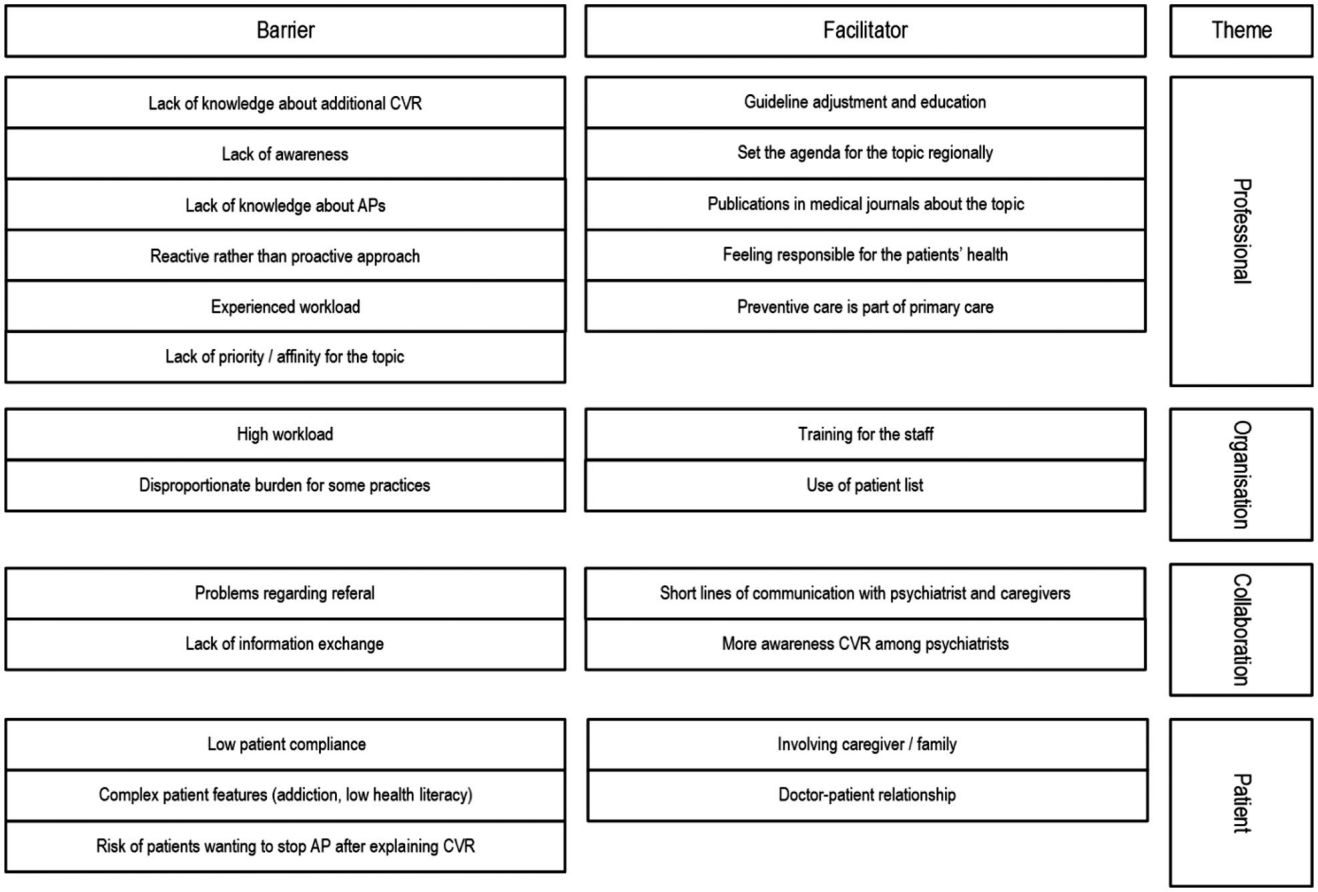

### Professional

The theme ‘Professional’ relates to the role of the GP as an individual.

All GPs reported a lack of knowledge on estimating the additional risk caused by SMI or AP use. Most GPs did not consider SMI an additional risk. The use of APs, however, was recognised by the GPs as increasing the risk of cardiovascular disease.

*I didn’t think she would qualify [for CVRM]. If she were interested, that would be fine but I really don’t think her risk is that high. She has bipolar disorder for which she has taken APs in the past. However, you state that she does have a higher risk? (GP 2)*

Neither SMI nor the use of APs is mentioned in the Dutch guideline for CVRM and the CVR calculation algorithm [22]; therefore, the GPs considered CVR-lowering medication unnecessary when the calculated risk was low (green) or moderate (yellow). Additionally, smoking cessation is the most effective measure, but all participating GPs assumed that this is difficult to accomplish for patients with SMI. Moreover, they indicated that they lacked expertise in the interactions of APs with tobacco, varenicline, or bupropion.

*I usually explain the risks by showing the risk chart. [For a] patient [who] smokes a lot…you can tell them ‘your risk is red, but don’t worry, we can do something about that.’ However, if you show them the chart and their risk is green…you can say ‘yes, your risk will be high in about 30 years,’ which is not very persuasive. (GP 1)*

GPs expressed varying degrees of awareness that SMI or using APs increases CVR. It was often overlooked.

*I think the importance [of this link] is known. You just need to make the connection at the moment of your patient’s visit… With rheumatoid arthritis, this [association] is already there, you see but now the connection ‘oh, SMI…[check CVR]’ needs to be held at the front of the mind. (GP 6)*

GPs emphasised that they need more education about AP side effects, pharmaceutical interactions and relevant patient factors to provide personalised care for these patients. According to the GPs, it would be helpful to regularly include this topic in medical journals and regional activities like the yearly benchmark.

GPs’ intrinsic motivation varied to provide adequate CVRM for patients with SMI or who use APs. All but two GPs provided responsive care instead of proactive care to patients with SMI or using APs; thus, these patients will only receive care if they ask for it.

*If we think ‘oh, this calls for immediate action’ then we’ll do that. As doctors, we don’t think in a very preventive way with these patients. We tend to act reactively. (GP 5)*

However, most GPs felt responsible for facilitating CVRM for (some) patients they recognised on the list.

*I want to implement [CVRM] right away because this group of people I feel involved with and responsible for… People with psychiatric disorders often fall through the cracks. The fact that APs have been prescribed shows something serious is going on, or they wouldn’t have had that medication. This gives us a certain responsibility. (GP 7)*

One GP admitted to feeling no affinity for psychiatry and consequently no motivation to invite these patients to CVRM.

*For me, this is niche. They [medical authorities] offload everything onto us. I’m not involved with this. Psychiatry is not necessarily my field of interest. (GP 4)*

Many GPs experienced a heavy workload when working with patients with mental health problems compared to other patients. The GPs in the urban practices with many patients of low socioeconomic status strongly linked the high workload to the feeling of discouragement about taking up CVRM. They expressed frustration about high consultation rates and a sense of being understaffed.

I instantly feel tired [looking at this list of patients]. Even more work to do. (GP 5)

Often GPs did not prioritise the invitation of patients with SMI and those taking APs to participate in CVRM programmes. They were preoccupied with various other topics (e.g. polypharmacy, elderly patients or renovating the practice). On the other hand, all GPs stated that preventive care is an important part of their work.

### Organisation

The theme ‘Organisation’ relates to the general practice organisation and its policy concerning CVRM. Most GPs found the opportunity to delegate the CVRM to nurses facilitating, and some indicated that their nurses responded positively to inviting this specific group of patients.

*The nurse said, ‘let’s give it a try and we’ll see how it goes’. (GP 1)*

Some GPs suggested the training of the nurses would be helpful by ensuring that they are well equipped with skills and knowledge. One GP thought his staff might be unable to cope with patients with SMI.

*I have a patient in mind who smells very bad…poor hygiene…lack of self-awareness. I imagine the nurses might be reluctant and think ‘what am I supposed to do here?’… They need to work one step at a time and take it slowly, with a lot of empathy. More skills might be required to improve [lower CVR] with this patient group. (GP 10)*

All GPs found the possibility of generating a list of patients with SMIs or those who use APs helpful, mainly because the list provided an overview of patients not receiving adequate care (see ‘Procedure’ in the Methods section). Most participating GPs needed assistance to generate it. Moreover, sometimes the list created extra work for the GPs because of errors in the electronic medical file.

GPs reported limited time as a major barrier to inviting patients with SMI or using APs to the CVRM programme. The length of our participants’ lists varied between 6 and 112 patients causing a disproportionate burden. The longer lists were found in practices located in deprived neighbourhoods and were an important barrier in the GPs’ decision on whether to invite the patients. Furthermore, with limited time, this is a task easy to postpone.

*You ask me why this has not been done. I think it’s very simple: if it’s not directly in front of me, it will remain on the to-do list. (GP 3)*

### Collaboration

‘Collaboration’ relates to the partnership between the GP, the psychiatrist and other mental healthcare providers. Most GPs found patients on the list for whom they predicted CVRM would be unachievable in their practice. The GPs were (yet) unsuccessful in referring these patients to a psychiatrist. Capacity problems of mental healthcare providers result in unstable patients not receiving adequate psychiatric care, despite the best efforts of GPs. The long waiting times for mental healthcare services were often mentioned, with GPs forced to bridge this gap.

According to the GPs, complex patients are challenging to refer. Most GPs believed that mental health providers accept patients with problems suitable to the offered therapy range. As a result, some patients with SMI who also suffer from addiction or intellectual disability rely on the GP for care.

Shorter lines of communication with a psychiatrist can be helpful here, as mentioned by one GP.

*We were so frustrated with mental healthcare organisation X that we contacted organisation Z, which is affiliated with home care. They have a very approachable psychiatrist, who now works in our building and walks in to see if we have new referrals or fill us in on patients. They only intervene for a short period, until the patient is stabilised, but from there, they are generally available for us if we need them. (GP 5)*

All GPs complained about not being properly informed by psychiatrists. Often, it remained unclear to them whether the psychiatrist screened for CVR; sometimes, it was even vague if mental healthcare had been completed.

*Well, if the psychiatrist is involved, it makes sense that he should do [the CVRM], but in my experience they never do, they don’t even ask us to do so. They don’t communicate. Nothing is mentioned about CVRM at all. Organisation X rarely writes a status update or a final report to me, so I don’t know whether or not they are treating my patient anymore. (GP 8)*

### Patient

The theme ‘Patient’ specifically relates to the GP’s thoughts and assumptions about patient factors influencing the risks of the intervention and the compliance of patients. GPs had many assumptions about patients with SMI and how CVRM would work out for them. They often considered these patients to be noncompliant and were incredibly sceptical about adherence for complex patients.

GPs were generally aware of the delicate balance in mental health of patients using AP. Some participating GPs feared patients might stop using AP because they had been made aware of the cardiovascular consequences.

Some GPs thought that patients would not be interested in CVRM because of the difficult circumstances in which they live. GPs noticed that problems in different areas of patients’ lives, such as poverty, substance abuse, or insecure housing, negatively influenced their motivation or capability to comply with CVRM.

*So if I start talking about…his cholesterol level…he will think, ‘What are you talking about? My GP doesn’t understand my struggles’. (GP 9)*

Furthermore, the GPs assumed that the patients with more complex SMI would be unable to control their lifestyle.

*You want the patient to be in charge and to get out more but of course, that is virtually impossible for patients with SMI. (GP 3)*

GPs suggested that printed information materials could raise patients knowledge of their CVR and reinforce the explanation given to them.

The involvement of family or other caregivers could facilitate CVRM for patients with SMI by supporting them with appointments and their healthier lifestyle or by revealing difficulties.

*Her daughter is registered in my practice too. She always comes along with her mother to appointments here. I usually know which family members are available. Maybe we should adjust this in our invitation letter: ask them to bring a family member or carer. (GP 10)*

Finally, according to the GPs, their relationship could be used as a tool to reach patients.

*He is homeless. Always dodging care. But if I ask him, he will do it. Yes, I have a bond with him. He is a charming person. (GP 7)*

## Discussion

### Main findings

This study identified several factors that may hinder or facilitate GPs in treating patients with SMI or using APs in a CVRM programme, which were divided into four themes. The main barriers were: underestimation of CVR for patients with SMI and ambivalence to apply risk-lowering strategies such as smoking cessation and medication prescription (professional); disproportionate burdens (organisation); poor information exchange between GPs and psychiatrists (collaboration); and scepticism about patient compliance, especially if their problems are more complex (patient). The main facilitators were feeling responsible for the patients’ health, the availability of a computer-generated list of patients, low thresholds for communication with psychiatrists, and the involvement of family/carers to improve patient compliance.

### Comparison with the existing literature

Implementing CVR estimation and CVR-lowering strategies in patients with SMI or using APs in the guidelines for CVRM can be beneficial. Other researchers previously mentioned the lack of clarity in guidelines as being an important barrier [12]. The updated European guideline recognises mental disorders as a risk modifier [6]. However, it still does not consider mental disorders in the suggested risk estimation models. Contrary to the QRISK3 tool [23], in which the cardiovascular risk related to mental disorders is better covered. Still, balancing the positive effects of APs on mental health on the one hand and the adverse side effects on CVR on the other hand remains a challenging task for the GP, especially when changes to prescribed APs are considered. A short line of communication with a psychiatrist helps obtain advice as one of our participants mentioned. Previously, Bramberg et al. [24] suggested the introduction of a liaison physician between GPs and psychiatrists, trained in internal medicine and somatic comorbidities of SMI.

The scepticism of our participants about expected adherence to appointments is in line with previous research [16]. One explanation is that it results from underlying negative stigma towards patients with SMI. Studies demonstrate that stigma creates barriers resulting in poorer physical care [17,25]. A key strategy for stigma reduction in healthcare is contact with trained people who lived the experience of a mental illness [26]. However, expecting low adherence is realistic to some extent, as a systematic review found that mental illness, addiction and low SES correlate significantly with not attending appointments [27]. Practical suggestions to improve compliance might help to implement CVRM. In line with other studies, some of our participants recommended involving supportive carers to improve attendance [16,24]. Different proposed strategies are direct methods such as telephone invitations or home visits, which are more effective than written invitations [16].

According to our participants, the patient list, which provides an overview of patients with SMI or using APs who lack annual screening, was very helpful. In the UK, there is a national register of people diagnosed with SMI or on lithium therapy [28]. With this register, Yeomans studied the effects of a template-based health check compatible with the primary care computer system. The system was used by 75% of the test region practices, resulting in more accurate data recording [29]. Similar to our finding that the length of the lists varied extensively between practices, the percentage of patients who were recorded in GP registers of SMI ranged from 0.5% to 1.5% (three-fold variation) for clinical commissioning groups in England [28]. Additionally, the association of socioeconomic deprivation with mental health disorders increases the workload of practices in deprived neighbourhoods [30].

### Strengths and limitations

The computer-generated list identifying suitable patients facilitates the GPs and has never, to our knowledge, previously been used as a tool during interviews with GPs. The list helped GPs illustrate their views and enabled the researcher to reflect on them, reducing the risk of researcher bias. The GPs were purposively sampled, and the participation rate was very high, providing a broad and diverse spectrum of the barriers and facilitators foreseen by GPs.

Our results depend on regional policies arranged by the primary care co-operative, which impedes direct generalisability. For instance, financial barriers might dominate the results in other regions of the Netherlands. Nevertheless, our results seem relevant to other regions or countries when planning the implementation of CVRM in patients with SMI or AP users. Furthermore, researcher KJ works for and with the GPs of the co-operative, which could have influenced their responses in the desired direction.

### Implications for practice

All CVRM guidelines should acknowledge mental disorders as risk modifiers and preferably instruct on how to estimate the additional risk.

Additionally, consultation opportunities with psychiatrists should be made available. GPs need advice if adverse metabolic effects worsen and if smoking cessation is considered during AP treatment. The availability of a computer-generated list of patients is interesting to use as a tool in interview studies. The interviewee can mention their barriers and facilitators before being influenced by the researchers’ questions.

Other preconditions that can be considered are support for practices in deprived areas and organising a stigma-reducing intervention with trained people who lived the experience of a mental illness for GPs and nurses.

## Conclusion

This study displays a range of barriers and facilitators anticipated by GPs into four themes. These indicate the preconditions required to facilitate GP inclusion of this specific population in CVRM programmes, namely adequate recommendations in practice guidelines, improved consultation opportunities with psychiatrists, practical advice to support patient adherence, and incentives for practices in deprived areas. Otherwise, CVRM for patients with SMI or using AP will probably remain on many to-do lists.

## Supplementary Material

Supplementary Material

## References

[1] Correll CU, Solmi M, Veronese N, et al. Prevalence, incidence and mortality from cardiovascular disease in patients with pooled and specific severe mental illness: a large-scale meta-analysis of 3,211,768 patients and 113,383,368 controls. World Psychiatry. 2017;16(2):163–180. 28498599 10.1002/wps.20420 PMC5428179

[2] de Mooij LD, Kikkert M, Theunissen J, et al. Dying too soon: excess mortality in severe mental illness. Front Psychiatry. 2019;10:855. 31920734 10.3389/fpsyt.2019.00855 PMC6918821

[3] Vancampfort D, Firth J, Schuch F, et al. Sedentary behavior and physical activity levels in people with schizophrenia, bipolar and major depressive disorder: a global systemic review and meta-analysis. World Psychiatry. 2017;16(3):308–315. 28941119 10.1002/wps.20458 PMC5608847

[4] Kivimaki M, Steptoe A. Effects of stress on the development and progression of cardiovascular disease. Nat Rev Cardiol. 2018;15(4):215–229. 29213140 10.1038/nrcardio.2017.189

[5] Domany Y, Weiser M. Insights into metabolic dysregulations associated with antipsychotics. Lancet Psychiatry. 2020;7(1):6–7. 31860456 10.1016/S2215-0366(19)30473-0

[6] Visseren FLJ, Mach F, Smulders YM, et al. 2021 ESC guidelines on cardiovascular disease prevention in clinical practice. Eur Heart J. 2021;42(34):3227–3337. 34458905 10.1093/eurheartj/ehab484

[7] De Hert M, Cohen D, Bobes J, et al. Physical illness in patients with severe mental disorders. II. Barriers to care, monitoring and treatment guidelines, plus recommendations at the system and individual level. World Psychiatry. 2011;10(2):138–151. 21633691 10.1002/j.2051-5545.2011.tb00036.x PMC3104888

[8] Jakobs KM, Posthuma A, de Grauw WJC, et al. Cardiovascular risk screening of patients with serious mental illness or use of antipsychotics in family practice. BMC Fam Pract. 2020;21(1):153. 32727372 10.1186/s12875-020-01225-7 PMC7391510

[9] Reilly S, Planner C, Hann M, et al. The role of primary care in service provision for people with severe mental illness in the United Kingdom. PLoS One. 2012;7(5):e36468. 22615769 10.1371/journal.pone.0036468 PMC3352919

[10] Magnee T, de Beurs DP, Boxem R, et al. Potential for substitution of mental health care towards family practices: an observational study. BMC Fam Pract. 2017;18(1):10. 28143421 10.1186/s12875-017-0586-4 PMC5282718

[11] Halfdanarson O, Zoega H, Aagaard L, et al. International trends in antipsychotic use: a study in 16 countries, 2005–2014. Eur Neuropsychopharmacol. 2017;27(10):1064–1076. 28755801 10.1016/j.euroneuro.2017.07.001

[12] van Hasselt FM, Oud MJ, Loonen AJ. Practical recommendations for improvement of the physical health care of patients with severe mental illness. Acta Psychiatr Scand. 2015;131(5):387–396. 25495118 10.1111/acps.12372

[13] McDonell MG, Kaufman EA, Srebnik DS, et al. Barriers to metabolic care for adults with serious mental illness: provider perspectives. Int J Psychiatry Med. 2011;41(4):379–387. 22238842 10.2190/PM.41.4.g

[14] Cramm JM, Adams SA, Walters BH, et al. The role of disease management programs in the health behavior of chronically ill patients. Patient Educ Couns. 2014;95(1):137–142. 24462120 10.1016/j.pec.2013.12.017

[15] Tjin A, Konings KTS. Revision Dutch guideline cardiovascular disease prevention 2019. Ned Tijdschr Geneeskd. 2019;163:D4237. 31483582

[16] Burton A, Osborn D, Atkins L, et al. Lowering cardiovascular disease risk for people with severe mental illnesses in primary care: a focus group study. PLoS One. 2015;10(8):e0136603. 26317516 10.1371/journal.pone.0136603 PMC4552729

[17] Ross LE, Vigod S, Wishart J, et al. Barriers and facilitators to primary care for people with mental health and/or substance use issues: a qualitative study. BMC Fam Pract. 2015;16:135. 26463083 10.1186/s12875-015-0353-3 PMC4604001

[18] Damschroder LJ, Aron DC, Keith RE, et al. Fostering implementation of health services research findings into practice: a consolidated framework for advancing implementation science. Implement Sci. 2009;4:50. 19664226 10.1186/1748-5908-4-50 PMC2736161

[19] Tong A, Sainsbury P, Craig J. Consolidated criteria for reporting qualitative research (COREQ): a 32-item checklist for interviews and focus groups. Int J Qual Health Care. 2007;19(6):349–357. 17872937 10.1093/intqhc/mzm042

[20] WONCA WOoFD. International Classification of Primary Care (ICPC-2). 2015. [cited 2017 Jan 5]. Available from: http://www.globalfamilydoctor.com/groups/WorkingParties/wicc.aspx.

[21] ATC classification index with DDDs [Internet]. WHO Collaborating Centre for Drug Statistics Methodology 2016/2017 [cited 2017 Jan 5]. Available from: https://www.whocc.no/atc_ddd_index/.

[22] Braun V, Clarke V. Using thematic analysis in psychology. Qual Res Psychol. 2006;3(2):77–101.

[23] Hippisley-Cox J, Coupland C, Brindle P. Development and validation of QRISK3 risk prediction algorithms to estimate future risk of cardiovascular disease: prospective cohort study. BMJ. 2017;357:j2099. 28536104 10.1136/bmj.j2099 PMC5441081

[24] Bjork Bramberg E, Torgerson J, Norman KA, et al. Access to primary and specialized somatic health care for persons with severe mental illness: a qualitative study of perceived barriers and facilitators in Swedish health care. BMC Fam Pract. 2018;19(1):12. 29316894 10.1186/s12875-017-0687-0 PMC5759233

[25] Corrigan PW, Mittal D, Reaves CM, `et al. Mental health stigma and primary health care decisions. Psychiatry Res. 2014;218(1-2):35–38. 24774076 10.1016/j.psychres.2014.04.028 PMC4363991

[26] Maranzan KA. Interprofessional education in mental health: an opportunity to reduce mental illness stigma. J Interprof Care. 2016;30(3):370–377. 27152542 10.3109/13561820.2016.1146878

[27] Dantas LF, Fleck JL, Cyrino Oliveira FL, et al. No-shows in appointment scheduling: a systematic literature review. Health Policy. 2018;122(4):412–421. 29482948 10.1016/j.healthpol.2018.02.002

[28] England P. Atlas of variation. Topic: Mental health disorders. Map 45 2015 [cited 2017 Jan 5]. Available from: https://fingertips.phe.org.uk/profile/atlas-of-variation

[29] Yeomans D, Dale K, Beedle K. Systematic computerised cardiovascular health screening for people with severe mental illness. Psychiatr Bull (2014). 2014;38(6):280–284. 25505628 10.1192/pb.bp.113.045955 PMC4248164

[30] Barnett K, Mercer SW, Norbury M, et al. Epidemiology of multimorbidity and implications for health care, research, and medical education: a cross-sectional study. Lancet. 2012;380(9836):37–43. 22579043 10.1016/S0140-6736(12)60240-2

