# Supplementary material for: Cardiovascular risk management in patients with severe mental illness or taking antipsychotics: A qualitative study on barriers and facilitators among dutch general practitioners
Source: Eur J Gen Pract. 2022 Jul 7;28(1):191–9. doi: 10.1080/13814788.2022.2092093 (PMC9272927; doi:10.1080/13814788.2022.2092093)

**Supplementary 1. Interview guide**

The patients’ list

Can you look up a few patients on the list in your medical files and indicate the main reasons why this patient was not included in the CVRM chain care? What could help facilitate the invitation of these patients for a CVRM screening?

CFIR-based questions

Intervention (of CVRM for patients on the list)

1. ***Supporting evidence*** What kind of additional information is needed about the increased CVR in patients with SMI/APs to get staff on board?
2. ***Benefits*** Does this approach have (dis)advantages over the current way care is arranged for them? If yes, which one?
3. ***Alternative*** Is there another intervention that you would rather implement? Can you describe that intervention? Why would you prefer the alternative?
4. ***Adaptability*** What changes or alterations do you think you will need to make to the intervention for it to work effectively in your setting? Do you think you will be able to make these changes? Why or why not?
5. ***Complexity*** How complicated is the intervention (inviting patients to the CVRM programme)? Please consider the following aspects of the intervention: duration, scope, intricacy and number of steps involved, and whether the intervention reflects a clear departure from previous practices
6. ***Design*** How do you find the quality of the supporting tools (patient list, invitation letter, document with regional agreements)? Do you know where to find the tools? Are they relevant? Are you missing anything?

Outer setting

1. ***Patient needs and resources*** How do you think patients respond to an invitation letter for CVRM screening? Have you heard stories about the experiences of others? Can you describe a specific story?
2. Are there characteristics of this patient group that complicate CVRM for patients with SMI/AP?
3. ***Colleagues*** Can you tell me what you know about colleagues who have implemented the intervention or other similar programmes? How has this information influenced your decision to implement the intervention?

Inner setting

1. ***Practice organisation*** How will your practice’s infrastructure influence the intervention’s implementation (practical implementation, maturity of the organisation, scope, current CVRM programme)? How will the infrastructure facilitate/hinder the intervention’s implementation?
2. ***Changes*** What kinds of infrastructure changes will be needed to accommodate the intervention? Changes in scope of practice? Changes in formal policies? Changes in information systems or electronic records systems? Other?
3. ***Meetings*** Are meetings, such as staff meetings, held regularly? Who typically attends? How often are the meetings held?
4. ***Culture*** To what extent are new ideas embraced and used to improve your organisation? Can you describe a recent improvement project?
5. ***Tension for change*** How essential is this intervention to meet the needs of the patients in your practice? How do people (including yourself) feel about current practice?
6. ***Compatibility*** Does CVRM for patients with SMI/AP fit with existing work processes? What are likely issues or complications that may arise? Will the intervention replace or complement a current programme or process? In which ways?
7. ***Priority*** Do you have ongoing projects with a high priority? What is the priority of getting the intervention implemented relative to other initiatives that are happening now?
8. ***Goals*** How does the implementation of the intervention align with other organisational goals?
9. ***Learning climate*** Can you describe a recent innovation in practice, including the motivation, milestones achieved, helping factors, key players, and your involvement? Were people happy with the outcome? To what extent do you feel like you can try new things to improve your work processes?
10. ***Resources*** Do you expect sufficient resources to implement and administer the intervention? If yes, what resources are you counting on? Are there any other resources you received or would have liked to receive? If no, what resources are not available?

Individual

1. ***Knowledge*** What do you know about CVRM for patients with SMI/AP or its implementation?
2. ***Beliefs*** How do you feel about the intervention used in your practice (stress, enthusiasm) and why?) How do you think things are going now?
3. ***Confidence*** How confident are you that you can implement the intervention successfully? What gives you that level of confidence (or lack of confidence)?
4. ***Internships of change*** (Show figure of Prochaska’s stages of change) Which phase represents your situation regarding the implementation of CVRM for patients with SMI or using AP?

Process

1. ***Planning*** Can you describe the plan for implementing CVRM for patients with SMI/AP? How detailed and realistic is it? Who knows about it? What is the division of tasks? What do those involved think of their role? Who is the leader?
2. ***Champions*** Other than the formal implementation leader, are there people in your organisation likely to champion (go above and beyond what might be expected) the intervention?
3. ***Key Stakeholders*** What steps have been taken to encourage individuals to commit to using the intervention? Who could need that?
4. ***Inviting patients*** Are you considering inviting patients to the CVRM programme differently?

Overall

1. ***Advice*** Do you have any additional remarks? Would you recommend this intervention?

Supplement Figure 1. Presentation of themes and categories


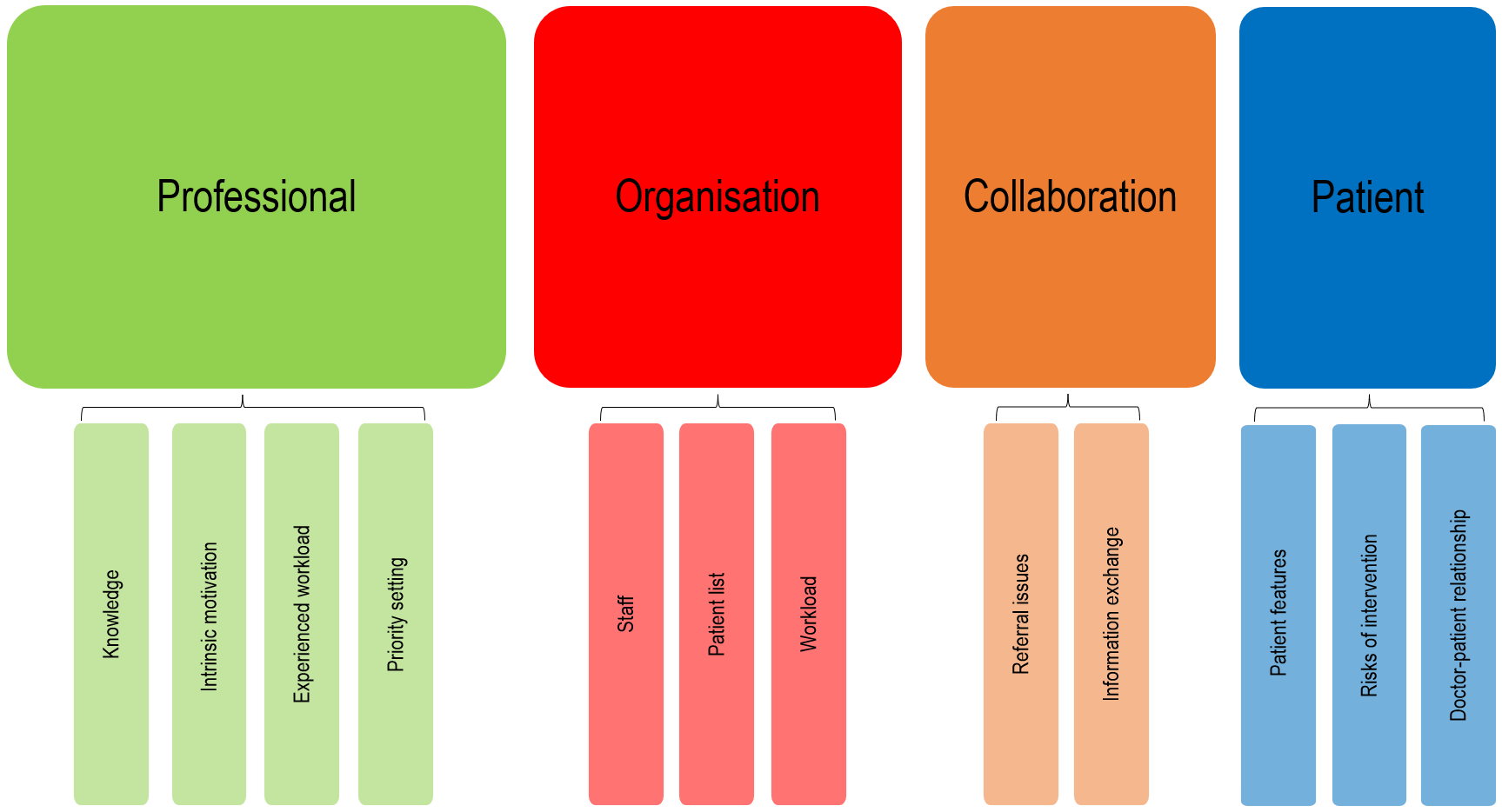

Supplement: Supplementary Material [file IGEN_A_2092093_SM2859.docx]
